# Qualitative assessment of rural health workers’ management of malaria in sick children

**DOI:** 10.5281/zenodo.10870159

**Published:** 2015-06-18

**Authors:** Ayodele S. Jegede, Ikeoluwapo O. Ajayi, Frederick O. Oshiname, Catherine O. Falade, Daniel Chandramohan, Hamade Prudence, Jayne Webster, Ebenezer Baba

**Affiliations:** 1 Department of Sociology, University of Ibadan, Ibadan, Nigeria; 2 Department of Epidemiology and Biostatistics, College of Medicine, University of Ibadan, Ibadan, Nigeria; 3 Department of Health Promotion and Education, College of Medicine, University of Ibadan, Ibadan, Nigeria; 4 Department of Pharmacology, College of Medicine, University of Ibadan, Ibadan, Nigeria; 5 Disease Control Department, London School of Hygiene & Tropical Medicine, London WC1E 7HT, United Kingdom; 6 Malaria Consortium, Development House, 56-64 Leonard Street, London EC24 4LT, United Kingdom; 7 Support for National Malaria Programme, Abia House, Abuja, Nigeria

## Abstract

**Background:**

Febrile illnesses are common causes of morbidity and mortality among under-five children in sub-Saharan Africa. The recommended strategy for effective case management of uncomplicated malaria is parasitological confirmation prior to use of artemisinin-based combination therapy (ACT). There is a lack of qualitative information explaining factors, which influence malaria case management practices among health workers. This study explores the perceptions of health managers and health care providers on the case management of uncomplicated malaria among under-fives in selected primary health care (PHC) facilities of two Local Government Areas (LGAs), Katcha and Gbako, as part of baselines for capacity-building interventions planned in Niger State, Nigeria.

**Methods:**

Interviewees included state- and LGA-level health programme managers, and frontline health workers purposively selected to cover a range of cadres involved in case management of sick children. Issues explored were history taking, diagnosis, appropriate diagnosis of malaria, prescription for malaria, referrals and adherence to referral. Data coding was carried out with Nvivo qualitative software (version 8) and content analysed.

**Results:**

History taking was often not carried out appropriately by the health workers. Treatment of malaria was not based on parasite-based diagnosis. Most of the health workers reported that they prescribed ACTs for treating presumed uncomplicated malaria. Care givers’ preferences, poor transportation systems and lack of financial resources led to poor adherence to referral advice. Absence of health workers from their duty post hindered effective service delivery. Prescription of ACTs as a first line of treatment for uncomplicated malaria without a parasite-based diagnosis was the standard case management practice.

**Conclusion:**

Parasite-based diagnosis for malaria will invariably lead to better treatment for non-malaria fever cases among the studied age group. Continuous capacity building aimed at improving adherence to current recommendations on parasite-based diagnosis and good clinical practice would be required to support the paradigm shift to parasite-based diagnosis of malaria.

## 1 Introduction

Effective health care services are needed to improve the health of the population [1]. Where this is not readily available, this often leads to poor management of common diseases like malaria. Health workers might not effectively treat fevers suspected as being malaria due to lack of proper diagnosis, and this might lead to complications resulting from misdiagnosis. Case definition of uncomplicated malaria within the context of the study is the presence of symptomatic malaria with parasitemia <5% without evidence of vital organ dysfunction, and the ability to take oral therapy [2]. Patients such as ‘young children, non-immune adults and immunocompromised with malaria may deteriorate rapidly’ [3] if treated wrongly or not treated at all.

Uncomplicated malaria is common in children below five years of age and is a major cause of fever in sub-Saharan Africa. Many cases of febrile illnesses are misdiagnosed, and mistreated or inadequately treated [4]. An estimated 90% of all malaria deaths occur in Africa, of which the majority are children under five (91%) [5,6]. Due to significant improvement in funds available for prevention activities directed at controlling malaria in Africa over the last decade, an 8.2% reduction in child mortality caused by malaria between 2001 and 2010 has been estimated [6].

The health system and health workers have important roles to play in addressing malaria control strategies [6,7]. Prompt access to malaria diagnosis and treatment are major components of the Roll Back Malaria Partnership’s Global Malaria Action Plan to reduce malaria morbidity and mortality by 2015 [1,8]. Studies providing evidence on how to improve access to health care services in low-resource settings, including for malaria case management, have been conducted, but appropriate treatment practice still requires better understanding of the drivers of health provider behaviour [1,9-13]. Effective case management of uncomplicated malaria entails proper clinical assessment, laboratory confirmation of the disease either by light microscopy or rapid diagnostic technique (RDT) prior to treatment with an effective antimalarial [2,14]. The Nigerian government changed the treatment policy for uncomplicated malaria from use of monotherapy-based to Artemisinin-based Combination Therapy (ACT) in 2005, in line with WHO recommendations. With the advent of quality-assured rapid diagnostic tests, accurate diagnosis of malaria is available at primary-level health facilities. Improved diagnosis leads to better management of sick children, whether or not they have malaria [2,14].

Health-seeking behaviour of primary caregivers for children with febrile illnesses follows alternate pathways in Africa, with the informal health sector usually being the first point of call [15,16]. In a study conducted in Ogun State, Nigeria, between 15% and 83% (median 50%) of child care providers visited medicine sellers at the onset of any illness [17]. An extensive review of empirical studies revealed that febrile patients are treated in many settings, including the home, drug shops, private clinics and public health facilities [15,17].

Human resource limitations, including inadequate training and shortage of staff, have been identified as barriers to effective case management of the sick child, including care involving malaria case management [18-22]. Prior to a recent policy shift on malaria case management, the Integrated Management of Childhood Illnesses (IMCI) guidelines had recommended the syndromic approach to define malaria cases among children at primary levels of care; however, recent WHO recommendation for case definition based on parasite-based diagnosis among all age groups at all levels of care constitute a significant shift in approach that have far-reaching implications on fever management at primary levels [14,21]. Current policy recommendations focus more on effective testing before treatment of uncomplicated malaria cases; this represents a shift from presumptive treatment [2,14]. This paper presents findings on health worker-related treatment practices for febrile illnesses pre-implementation of a renewed malaria case management capacity-building programme in Niger state. The study was conducted when the revised national malaria policy was in the process of translation to practice with mRDTs being rolled out at the primary level of care.

## 2 Methods

### 2.1 Study aim

The study was exploratory in nature using qualitative techniques of data collection. The aim of this study was to provide qualitative explanation of factors influencing case management of uncomplicated malaria among under-five children in the study area.

### 2.2 Study area

This study was carried out in Niger State, Nigeria. The state has 25 Local Government Areas (LGAs) and it lies in the North Central geopolitical zone of Nigeria. Infant and under-five (U5) mortality rates are high in Niger State at 85 and 106 per 1000 live births, respectively, which is very close to the national average of 75 and 106 per 1000, respectively [23]. Malaria is endemic in the state with perennial transmission. At the national level, only 28.9% of U5 were reported to have slept under an insecticide-treated net (ITN) nationwide in 2010 [24]. Net use in Niger as of 2009 was 31.1%, a prevalence higher than the national and regional averages [24].

Within the 25 LGAs in the State, there are 1,323 Primary Health Care (PHC) facilities, 18 Secondary Health facilities and two Tertiary health facilities, as well as 446 registered Private Health facilities. In addition, there are 1,200 licensed Patent Medicine Vendors. All PHC facilities are under the Primary Health Care unit of each LGA, headed by the PHC coordinator, who could be a medical doctor or environmental health officer. The PHC facilities are mostly open from 8 am to 4 pm; a few are designated to provide 24-hour services, but do not, however, function as such.

The majority of the health workers in the two LGAs were Junior Community Health Extension Workers (JCHEWs) and Community Health Extension Workers (CHEWs). Only one doctor was available in Katcha. These cadres of health workers were also responsible for providing health care services for sick children. The majority of the health facilities were headed by CHEWs. Many of the facility-based health workers had worked for more than three years in the health facilities.

The PHC facilities participated in the distribution of ITNs to pregnant women and mothers of U5; they also provided family-planning services as well as conducted health education relating to hygiene and nutrition. Artemether-Lumefantrine and Artesunate-Amodiaquine are provided for free to U5. The health facilities are provided with malaria commodities by the state central medical stores and development partners. Before the study commenced in late 2011, the malaria case management capacity building process had already commenced in some other LGAs of the Niger State.

### 2.3 Sampling and study procedures

The study was carried out in November and December 2011 in PHC facilities in Katcha and Gbako LGAs. These LGAs were purposively selected for the assessment of the State malaria case management capacity building programme and were matched in terms of ratio of public:private sector facilities and socio-economic status. A multistage sampling strategy was used to select the study health facilities. The health facilities were first stratified into public or private and then into secondary or primary health facilities. The number of health facilities sampled from each of these categories was proportional to the size of each category. Where the number of secondary health facilities was less than three, all were included; the six health facilities included in the study were among the 14 selected for the malaria case management capacity-building intervention

The health workers interviewed were purposively selected to cover a range of cadres involved in malaria case management. The issues explored in the interviews included history taking, diagnosis of malaria, dispensing of malaria treatments, management of associated or alternative conditions, referral of severe cases and adherence to referral, interpersonal communication and training.

The study team and the Roll Back Malaria coordinator visited the prospective respondents at least a day before the interviews to brief them about the study and the request for their participation. The interviews were conducted in English, as the respondents could speak, understand and were comfortable in this language. A research assistant with expertise in public health and sociology, assisted by a note taker, conducted the interviews. The note taker both monitored the tape recording and manually recorded the key issues and observations (verbal and non-verbal events) of the interviewees. The interview guides used were unstructured and all questions were open-ended. The interviews were flexible, allowing exploration of emergent issues that were not in the original topic guide but were raised by the interviewees.

### 2.4 Data coding and analysis

The interviews were transcribed, read and re-read in order to understand the data. After this familiarisation with the data, the interviews were transferred to Nvivo 8 software for coding. Codes were developed and finalized by a single coder. The coding process followed an integrated approach, making it both deductive and inductive. The initial phase of coding was largely deductive, as study objectives served as the bases of coding as well as titles of codes (nodes) in ‘tree nodes’. For instance, tree nodes were created to accommodate all data about attendance at an IMCI workshop. ‘Coding on’ was accomplished following an inductive tradition. Hence, content of initial nodes mainly determined the codes and code structure that were further developed. For instance, child nodes created from the tree node of ‘challenges faced at health facility’ were titled ‘shortage of drugs’, ‘shortage of staff’ and ‘lack of accommodation’. These three nodes, along with others, made up the content of the tree node. Hence, an initial code could eventually be broken down to a fourth generation, although this was largely not the case. Most nodes were not broken down at all; some were broken just once, and some twice. ‘Free nodes’ (stand-alone nodes) were also created for data yet to be made sense of. A single person performed the coding of transcripts of interviews conducted and transcribed by the interviewers [25-27].

All interviewees were assigned anonymous health worker numbers, so that neither roles, levels within the healthcare system nor health facilities could be identified in order to maintain confidentiality.

### 2.5 Ethics

The focus and nature of the in-depth interviews were introduced to participants within the context of the baseline quantitative study for the malaria case-management capacity-building evaluation. The objectives and procedures of the study were explained to the officer in charge of the health facility and a verbal consent was obtained. A list of all staff involved in diagnosis and treatment of sick children and their roles were obtained for each selected health facility. A meeting was held with all health facility staff, and the study objectives and procedures were explained. Eligible staff members who were not present at the meeting were approached individually to obtain their consent. Written informed consent was obtained from carers and health care providers prior to the interview. Approval of the study protocol was obtained from the Ethics Committees of the Niger State Ministry of Health, the University of Ibadan/University College Hospital (IUI/UCH) and the London School of Hygiene and Tropical Medicine.

## 3 Results

Sixteen in-depth interviews were conducted. This comprised two key informant interviews at the state level with the PHC Director and the Monitoring and Evaluation Officer. In each of the two LGAs, interviews were conducted with one LGA-level Roll Back Malaria Manager, one Monitoring and Evaluation Officer, one PHC Director, one LG Chairman and three health workers. The health workers were either Community Health Officers (CHO), Community Health Extension Workers (CHEWs) or Junior Community Health Extension Workers (JCHEWs). Most of them had worked more than three years in their health facilities and received trainings from other development partners.

### 3.1 Clinical diagnosis

***Reasons for using clinical diagnosis***

Presumptive treatment was commonly practised in the study area, this may be as a result of lag in the implementation of policy to promote parasite based diagnosis before treatment in all age groups which had been changed almost one year earlier as at the time of the study, RDTs had not been fully rolled out to all health facilities. The reasons for using presumptive methods of diagnosis include a lack of laboratory facilities, lack of RDTs diagnosis and lack of malaria diagnosis-related training. According to respondents within the LGAs.

“*We base treatment on what patient tell us. We do not have facilities to test patient. This is how patients are being treated here.*” [IDI A, Katcha LGA]

The non-availability of RDTs was mentioned as an acute problem facing diagnosis and treatment of non-complicated malaria by most of the respondents. Respondents said that a non-governmental organisation (NGO) provided support to training and implementation of malaria case management, but that the Ministry of Health and Partners were yet to supply RDTs to all health facilities in the state. Capacity-building efforts in the selected LGAs aligned to new policy guidelines from the Federal Ministry of health on parasite-based diagnosis (2010) were yet to commence in the study sites as at the time of the survey:

“*Since SUNMAP (a development partner) started supplying us ACT, they promised that they will supply RDT but up till now RDT kits are yet to be supplied. So, to do malaria test is difficult. We only treat fever in time; we assume it is malaria so that we give them ACTs.”* [KII A, Gbako LGA]

From time to time, National Malaria Control Programme (NMCP) and partners supplied the LGAs with ACTs as a supplement to the Government supply. Only health workers in the LGAs that had received training on case management also received RDTs; health workers yet to receive such training were not supplied with RDTs. An in-depth interviewee at the state level explained the situation thus:

“*Since the training has not covered everywhere, most children are being treated presumptively. Once a child comes to the health facility with fever, he/she is given ACT. But at the LGAs where health workers have been trained, they tend to send those patients for test to confirm the actual diagnosis. Based on the result they would be given appropriate treatment.”* [IDI F, State level]

***Clinical diagnosis in practice***

Most health workers stated that they relied on algorithms, clinical experience, signs and symptoms for making presumptive diagnosis of malaria. There was consensus among the health workers on the utility of treatment manuals and standing orders in decisions making on diagnosis in patients with non-complicated malaria. According to a respondent:

“*Since we do not have any functional laboratory for confirmatory test, we simply use the Standing Order [treatment manual] for malaria to guide treatment decisions as diagnosis is based on observed signs and information provided by mothers or whoever brings the sick child.”* [KII B, Katcha LGA]

Past experience, based on many years of working in the locality, and therefore having developed knowledge of clinical signs and symptoms for the common diseases, was an important contributing factor among health workers reinforcing their constituted an important source of confidence on diagnosis. An in-depth interviewee declared:

“*People learn on job one has been doing for years. Experience is important in medicine and because we are familiar with the common diseases affecting children in this community it is easy for us to treat them with signs and symptoms.”* [IDI H, Katcha LGA]

The reported most important sign for clinically diagnosis of malaria based on the responses of the health workers was that of high temperature.

*“*If *we have febrile children we have to first take the temperature with clinical thermometer to know the degree of hotness of the body. So after that we soak towel inside water to cool the temperature so that temperature will come down. After the temperature might have come down then we give antimalarial and possibly any analgesic drug so as to calm the temperature.”* [IDI B, Katcha LGA]

“*Normally, if a patient having cold or his/her body is hot with temperature as high as 38°C, then we know that the child has malaria. But there is no way we can confirm that the observation is correct. It is subject to individual’s* [health worker’s] *experience.”* [KII D, Katcha LGA]

Whereas some clinicians used thermometers to measure temperature, others determined patients’ temperature by placing their hands on the patients. According to a respondent:

“*When a patient comes, I use my hand to feel if the body temperature is hot or not. Through this I will be able to know if the body temperature is high or not. I have been doing this for a long time and many health workers also do so In fact, this is what people do at home too.”* [KII B, Katcha LGA]

This practice suggests a combination of appropriate and unorthodox techniques of assessments. Although subjective and lacking standardization, this method for assessing temperature was widely used even when thermometer is available.

While a raised temperature was considered a sign of malaria, the level to which the temperature was raised helped the health worker to decide upon the immediate course of action to take or recommend to the child’s carer.

“*I do not give ACT immediately because some children may just be running fever. What I do sometimes is to observe such a child for one day, especially when the body temperature is mild. But when the temperature is high I treat for malaria and give ACT.”* [IDI I, Gbako LGA]

Together with temperature, the health workers interviewed placed great emphasis on their diagnosis on the reports of the child’s symptoms from the carer. Symptoms considered indicative of malaria included vomiting, sleeplessness, and restlessness.

“*In the absence of a laboratory facilities for malaria test, I base my diagnosis on the signs and symptoms the mother of the child mentioned during consultation. Some of these include vomiting, high body temperature, restlessness and sleeplessness.”* [IDI J, Katcha LGA]

Similarly, another respondent corroborated this view in Gbako LGA.

“*Many health workers in this area recognise when a child is sick. We watch out for things like lack of sleep, restlessness and body temperature. When these signs are present, an experienced health worker will know that the child needs to be treated for malaria.”* [IDI D, Gbako LGA]

Some of the health workers were comfortable with presumptive diagnosis as it can be correctly used to diagnose malaria. But they would still like to have access to testing.

“*We don’t have laboratory in this facility. We only diagnose with signs and symptoms the child present with. That is how we have been doing it and it works. But it will be good if we have the test kits to work with.”* [IDI G, Gbako LGA]

There were two concerns expressed with the outcome of presumptive diagnosis from the perspective of the health workers interviewed, these were determining which fevers suspected as malaria were non-malaria fevers. The second was avoiding the overuse of drugs in patients for whom they were not necessary. A key interviewee declared:

“*Once a child presents with malaria-related signs or symptoms, we treat fever instead of malaria, because we don’t have laboratory facilities to confirm if it is malaria or not. Sometimes, one may treat non-malaria as malaria and this is dangerous especially because patients will use drugs they needed not to use.”* [KII C, Gbako LGA]

Laboratory services are occasionally required for diagnosis, especially when health workers are uncertain and want to be specific about prescription especially in situations of recurring illness. Respondents said that patients might be asked to go for malaria test outside the health facility to be able to ensure proper diagnosis for appropriate treatment to be given, particularly when they were worried that the patient might develop complicated or severe malaria. According to a key respondent:

“*Sometimes we ask patients to go to outside laboratory/ facility and do the malaria test. This is important because complicated cases are difficult to determine presumptively. One needs to do parasitological test to determine cases appropriately; this will make prescribing easier.”* [KII E, Gbako LGA]

### 3.2 Treatment for malaria

Depending on the diagnosis, health workers decide which drug to prescribe with some suggesting that this was based upon national recommendations. Use of standing orders was mentioned by some of the health workers. According to most the health workers who responded to the IDIs, they did refer to standing order in the treatment of patients. An interviewee summarised the situation thus:

“*We have a Standing Order serving as a guideline which aids our diagnosis and treatment of patients. We got the Standing Order from the local government authorities.”* [IDI B, Gbako LGA]

Many of the interviewees stated that ACTs were in fact prescribed by many of the health workers in both LGAs. A typical response was as follows:

“*Here in this facility, we dispense ACTs to patients to treat malaria because this is what is recommended to be given when malaria is diagnosed.”* [KII F, Katcha LGA]

“*For malaria treatment, I usually prescribe artesunate-amodiaquine (ACT) and vitamins B and C.”* [IDI, A Katcha LGA]

The in-depth interviewees threw some light on the artemisinin therapies that they provide. These therapies included monotherapies. Their usage pattern of artemisinin-based therapies are summarised in the following statement by an interviewee:

“*At times, when we don’t have the ACT we prescribe and dispense artesunate-amodiaquine (ACT).”* [IDI N, Katcha LGA]

“*We use drugs like CoArtem, artesunate tablet or artesunate syrup. If we want to give injection, we prescribe something like paracetamol injection. We also use artemether to calm the temperature.”* [KII F, Katcha LGA]

The key respondents prescribe a wide range of medicine for the care of sick children. These medicines included antibiotics, antipyretics and even chloroquine, which is no longer promoted for the treatment of malaria in Nigeria. This prescription pattern is inherent in the following statement:

“*I prescribe other drugs to complement ACTs. These other drugs include paracetamol and amoxicillin. At times, some patients request for chloroquine, which I prescribe for them to go and buy.”* [KII G, Katcha LGA]

Similarly, a respondent said:

“*What I do is that I prescribe ACT and depending on the condition of the child, I will add another drug, most especially paracetamol and antibiotics. When some people demand for Chloroquine I did prescribe it for them but we do not dispense it here because we do not have it.”* [IDI K, Gbako LGA]

CoArtem was predominantly distributed by the government and development partners pre-packaged, according to age categories. The brand of AL distributed was CoArtem (Novartis Pharma) because this brand was the first to be pre-qualified by the WHO, and Novartis offered it at a no-profit cost. According to an interviewee:

“*The ACT drugs are pre-packed according to age categories and usually taken for three days.”* [IDI L, Gbako LGA]

A few of the respondents revealed that they prescribed according to the weight of children of the child for most drugs.

“*We use the weighing scale to check the weight of children from 0 to 1 year. So, the accurate weight we get from the scale determines what dose to prescribe for the child. For example, if you ask them how many years is your child now, some will say three years for a one year child because many of the parents do not know the actual year they gave birth to their children. Because of this, we usually ask the child to climb onto the scale for weighing.”* [IDI M, Katcha LGA]

### 3.3 Referral

Reasons for referral of children to other health facilities, usually hospitals, included complicated cases, high temperature and lack of improvement after a three-day review; and treatment without signs of recovery. Complicated cases were said to be children presenting with more than one symptom, high body temperature and vomiting. Complicated malaria is described thus:

“*When they bring a child, I first check the temperature to know whether the child has malaria or not. But occasionally there may be children who will be vomiting. When I see this type of case I quickly send them to hospital.”* [IDI K, Katcha LGA]

“*I usually emphasize that if the signs and symptoms of the children persist after three days of being administered with the drugs, they should bring their children and if they come back here with such case and I see no sign of improvement, I do refer them without delay to bigger hospitals such as the General Hospitals in Minna and Bida.”* [IDI K, Katcha LGA]

Health workers stated that a child that becomes sick after he or she had been treated was referred to the hospital. They had indicated that treatment at the health centre might sometimes not be effective due to lack of proper diagnosis. According to the health workers, it is not good to keep on treating such a child; rather, they should be referred for proper diagnosis and treatment in the hospital. A typical response was as follows:

“*I do refer patients to general hospitals for better treatment whenever the diagnosis is inconclusive, especially when the child has a persistently high body temperature regardless of all efforts to reduce it. Most of the time, I refer them to General Hospital in Bida.”* [KII I, Katcha LGA]

While believing, as above, that children should be referred to healthcare facilities with diagnostic equipment, health workers in some health facilities were sceptical about doing this, as they did not believe that the children would be taken to the place of referral due to long-distance travel. The interviewees were of the opinion that travel time was a determinant of adherence to referral advice. This was also influenced by the availability of transport and type of transport available. An interviewee explained thus:

“*There is no reason to refer people to hospital here because they will not go. This is because the nearest hospital is far away and there is no transportation. But when the condition becomes serious some people may decide to go to where they are referred or elsewhere.”* [IDI R, Gbako LGA]

“*Patients are usually concerned about the distance of place of referral. For instance, it takes an average of one hour and thirty minutes to get to Minna and Bida, respectively, from this health facility in Bisanti. How long it takes to get to these two places however depend on how soon one gets a vehicle. Also, the type of vehicle one gets to board may determine how soon one will get there.”* [IDI S, Katcha LGA]

“*Our patients suffer here when they are referred to higher hospitals because we don’t have mobile referral service. Many people may even die on the way because of lack of transport. If we have our own transport here we can easily convey them to the appropriate hospital where we refer them to.”* [IDI T, Gbako LGA]

“…*In Niger state, you must have gone round and see that the state is big. Niger state occupies 10% of this country’s land mass. The land mass is so wide. So, you need a lot of resources to meet the challenges of logistics for health care delivery especially referral. Even if one decides to use private vehicle, this requires funding for maintenance and fuelling of the vehicle. The Monitoring and Evaluation unit needs a lot of resources to do good monitoring. Therefore, logistics is number one thing that affects referral service.”* [KII A, State Office]

Lack of adherence to place of referral was also seen as being due to patient or carer choice particularly with respect to attending private rather than public health facilities.

“*At times when I give the mothers the referral letter to take their children to the General Hospital in Minna, some will rather take the letter to a private hospital.”* [IDI K, Katcha LGA]

Various medications were reportedly used to treat patients with malaria, especially when desired ones were unavailable. According to an interviewee:

“*At times, when we don’t have the ACT we prescribe and dispense artesunate-amodiaquine (ACT). If we don’t have ACTs or artesunate in stock, we simply prescribe and ask our patients or their guardians to go and buy the drug at Kataeregi.”* [IDI N, Katcha LGA]

### 3.4 Other health system barriers

Health system barriers to appropriate treatment practices included cost of drugs, stock-outs of drugs and inadequately staffed health facilities. Health workers perceptions of the affordability of drugs to the child’s carer influenced their prescribing behaviour. This included the type of antibiotics prescribed alongside ACTs. The contribution-prescribing behaviours among the interviewees can be gleaned from the following statement:

“*In addition to the antimalarial drug prescribed and dispensed, I sometimes prescribe and dispense antibiotics such as ampiclox and ampicillin, which the mothers and other patients find cheaper and affordable to buy. These antibiotics are very effective regardless of their low prices.”* [IDI O, Gbako LGA]

The cost of ACTs to patients in the public sector health facilities varies due to their source. When supplies from development partners are out of stock, ACTs must be bought in the local private market where they are more expensive. It was interesting to observe that the health worker felt that it was necessary to add a mark-up when making prescriptions relating to ACTs that would be bought in the private market. A key informant eloquently described the practice as follow:

“*The challenge I face is in terms of the private sector because nothing is free in private sector and not all our caregivers can afford to pay. Sometimes I have to remove certain things so that I can be able to cut cost. Sometime we may put the cost of ACT at N100.00 when we ran out of stock because of supply from the SUNMAP (Partners). Then I have to go to the market to buy if I want to continue to treat them. The cost may be N80.00 in the market and if I dispense it at N100.00 they may not be able to afford it. Occasionally, when we asked them to pay N300.00 for CoArtem, paracetamol with other antibiotics put together some may not be able to afford it still. Occasionally, I have to write it for them to go and buy outside*.” [KII H, Gbako LGA]

Regularity of staff in their duty post was considered to be a factor that influences quality of service delivery, especially access of patients to treatment with ACTs. Some respondents reported that whenever a staff member was absent from his or her duty post, patients would not be able to receive treatment. The interviewees noted that rural communities in the state were served mainly by primary health care facilities, and most of them have only one or two qualified health workers. According to a health worker who just resumed in a health facility:

“*The problem before I was posted here was that the health worker does not live here. Whenever she is not around the facility will not be opened. As you can see now I am the only one here and I am doing a course in the city. Whenever I am not able to come there is no way this place will be open. It is a great challenge.”* [IDI P, Gbako LGA]

## 4 Discussion

Health workers are trained to treat patients, using national guidelines based on appropriate techniques, skills and standards of care. Treatment practice determines, to a large extent, the quality of care received by patients on many occasions. End users’ health-seeking behaviour also influences treatment practices of health workers. Effective treatment of uncomplicated malaria involves appropriate diagnosis and prescription using the national and WHO guidelines. Factors influencing practice extends beyond training of health worker to other health system barriers. All of these have to be taken into consideration as part of efforts to implement new policy recommendations. The alignment of interventions to address the identified health system barriers is a critical success factor for effective policy change. This study examined health workers’ perception of case management of febrile illness in rural health facilities.

Generally, effective management of uncomplicated malaria in under-five children in rural health facilities should be consistent with the treatment guidelines of the national malaria control programme and the WHO [2,28]. This study revealed that treatment practice is not consistent practice with the recommendations [26,27]. Information captured during history taking and examination as part of fever management should probe for uncomplicated and severe malaria, and should help to determine the presence or absence of concomitant or alternative infections [29].

While most of the health workers used presumptive diagnosis before treatment, a few of them indicated that they carried out parasitological tests using the mRDTs and some of them referred patients for diagnosis in local private laboratories. Generally, presumptive diagnosis and treatment was mostly used for management of malaria, as no parasite-based diagnosis was available in many areas [30]. The effectiveness of the presumptive treatment is limited because the symptoms and signs of malaria are non -specific and overlap with those of other febrile illnesses. Findings from sentinel sites during the 2009/10 drug therapeutic efficacy studies for management of uncomplicated malaria showed that, during the study period October 2009 to November 2010, of the 5,217 under five-year-old children with symptoms suggestive of acute uncomplicated malaria screened, parasitaemia was present in 1826 (a slide positivity rate of 35%). Rates ranged from 19% in the northcentral (NC) study sites (at relatively higher altitude of the northcentral plateau region) to 56% found in the mangrove south (SS).

Overall, parasite rates in northern sentinel sites (Northcentral, Northeast and Northwest) were significantly lower than in the southern sentinel sites (Southeast, South-south and Southwest regions) of the country [14]. The implications of presumptive treatment for uncomplicated malaria might be the gross over diagnosis and inappropriate administration of antimalarial drugs for non-malarial febrile illnesses. It could also mean that cases of co-infection are missed, delays occur in diagnosis of other causes of febrile illnesses, with resultant delay in instituting appropriate management with potentially dire consequences for children less than five years of age [31-33].

A very important consequence of presumptive treatment of malaria is the public perception that all fevers are malaria, and the implication of limited capacity for primary health facilities to diagnose and provide appropriate treatment options for non-malaria fevers would continue to exert pressure on health workers at the primary levels of care to treat all fevers presumptively as malaria. The persistence of monotherapies, some of which have antipyretic properties, further affects the mindset and choices of healthcare givers in light of observed temporary relief. An effective paradigm shift to parasite-based diagnosis and treatment for malaria thus needs to include a careful reflection on capacities and competencies to address treatment option for parasite-negative fevers.

The study also indicated that most children were treated without parasite-based confirmation of diagnosis because, at the time of the study, they had limited lab facilities for microscopy and little or no RDTs available. The low rate of laboratory diagnosis of malaria noted in this study is similar to a hospital-based clinical audit report in a children’s ward in Malawi [34]. The National Guidelines for diagnosis and treatment for malaria had been revised in March 2011 to strengthen emphasis on parasitological confirmation for diagnosis and treatment of malaria in all age groups [28]. The presence of private and secondary health facilities was minimal in the study area due to the largely rural setting. The use of RDTs was low despite the fact that this is currently being promoted as an acceptable alternative to microscopy in poor resource setting [26,33]. The introduction of more widespread parasite-based diagnosis of malaria opens further questions on cost effectiveness of current fever management strategies in resource-poor settings, which needs further exploration.

Although most of the health workers indicated that they prescribed ACTs, none of them reported that they based decision to make prescription on weight assessments, because they relied more on age as, reflected in treatment algorithms. As both age and weight are important factors in determining appropriate dosage of the recommended first-line treatment ACTs, failing to use these parameters might lead to the use of inappropriate dosage of these drugs. Although it has been argued that this has simplified the determination of correct dosage of antimalarial and improved prescription pattern among health care providers in the absence of weight measurement [4,31], ineffective communication of this important information to caregivers might result in poor adherence to treatment regimen and incorrect use of appropriate dosage of the drugs. A study conducted in Nigeria reported that inadequate evaluation and underreporting of clinical findings were more common in the private than public health facilities [32]. It has been argued that, as the ACTs are currently the first-line treatment for uncomplicated malaria, effort needs to be made to prevent them from being wrongly prescribed [4]. Aside from wrong prescription, substandard drugs can be prescribed. A study [16] has shown evidence of use of non-recommended drugs like chloroquine (CQ) in health facilities in the treatment of uncomplicated malaria.

Not all of the health workers interviewed indicated that they provided referral advice, and their perception was that only a small proportion of the caregivers complied with the referrals. The low rate of adherence to the referral advice raises a serious concern. It was also clear from the interviews that health workers referring patients often did not see the outcome of the referral. Caregivers did not like to be referred to other health facilities because of long distances, poor transportation system and lack of financial resources to afford the cost of transportation in the study areas.

This study had some limitations. There was limited number of private and secondary health facilities in the study area to compare their practices with those of the primary health facilities. This was because the study locations were rural in nature. Generally, rural areas are not attractive to private sector investors in health care delivery. Thus, the government also does locate public secondary health facilities in urban rather than rural areas, probably due to available supporting infrastructure, like road networks, pipe-borne water supply and electricity. As a result, it is not clear how service delivery in respect of case management of uncomplicated malaria compares in these categories of health facilities. Second, given the qualitative nature of this study, the results cannot be generalized and are therefore limited to the study site.

## 5 Conclusions

Most of the health workers prescribed ACTs when they were available or wrote prescriptions for ACTs to be prescribed elsewhere. Health workers did not seem to realise that ASAQ was an ACT and health workers also prescribed CQ and other non-effective antimalarial drugs if patients requested them as a first-line treatment for uncomplicated malaria. Most uncomplicated malaria cases were still being diagnosed presumptively as the existing standard practice and there were shortfalls in laboratories and mRDTs for perform parasite-based diagnosis. Beyond commodities and training, other health system challenges existed that could impact on uptake of policy recommendations, which would need to be taken into account in the operationalization of policy recommendations.

Taking the advantage of the new guideline, the strategy for implementation needs to take on board health provider-related and health-seeking behaviour of caregivers of children. Health worker training needs to incorporate a stronger focus on how to use RDTs for malaria diagnosis, interpersonal communication and options for treating non-malaria fevers in resource-poor setting. In addition, health facilities need to be supported to make timely parasite-based diagnostic services available at all times. Apart from the fact that this will improve case management of uncomplicated malaria, it will provide an opportunity to strengthen overall fever management. Referral mechanisms need to be strengthened to ensure that non-malaria fever cases receive appropriate treatment. Training and supportive supervision of health workers on the use of the national malaria treatment guideline would ultimately improve rational use of antimalarial drugs.

## References

[1] Kizito J, Kayendeke M, Nabirye C, Staedke SG (2012). Improving access to health care for malaria in Africa: a review of literature on what attracts patients.. Malar. J..

[2] World Health Organization. (2010). Guidelines for the treatment of malaria..

[3] Warsame M, Hassan AM, Barrette A, Jibril AM Treatment of uncomplicated malaria with artesunate plus sulfadoxine-pyrimethamine is failing in Somalia: evidence from therapeutic efficacy studies and Pfdhfr and Pfdhps mutant alleles.. Trop. Med. Int. Health..

[4] Leslie T, Mikhail A, Mayan I, Anwar M (2012). Overdiagnosis and mistreatment of malaria among febrile patients at primary healthcare level in Afghanistan: observational study.. BMJ..

[5] Pasquale H, Jarvese M, Julla A, Doggale C (2013). Malaria control in South Sudan, 2006–2013: strategies, progress and challenges.. Malar. J..

[6] Eisele TP, Larsen DA, Walker N, Cibulskis RE (2012). Estimates of child deaths prevented from malaria prevention scale-up in Africa 2001-2010.. Malar. J..

[7] Rao VB, Schellenberg D, Ghani AC: (2013). Overcoming health systems barriers to successful malaria treatment.. Trends Parasitol..

[8] Roll Back Malaria Partnership: Global Malaria Action Plan For a malaria-free world Geneva: (2008). World Health Organisation;.

[9] Hausmann-Muela S, Ribera JM, Nyamongo IK: (2003). Health-seeking behaviour and the health system response London: London School of Hygiene & Tropical Medicine..

[10] Obrist B, Iteba N, Lengeler C, Makemba A (2007,). Access to health care in contexts of livelihood insecurity: a framework for analysis and action.. PLoS Med..

[11] Hetzel MW, Iteba N, Makemba A, Mshana C (2007). Understanding and improving access to prompt and effective malaria treatment and care in rural Tanzania: the ACCESS Programme.. Malar. J..

[12] Rutherford ME, Mulholland K, Hill PC: (2010). How access to health care relates to under-five mortality in sub-Saharan Africa: systematic review.. Trop. Med. Int. Health..

[13] Ricketts TC, Goldsmith LJ: (2005.). Access in health services research: the battle of the frameworks.. Nurs. Outlook,.

[14] Federal Ministry of Health, National malaria control programme, Abuja, Nigeria. (2009). Drug therapeutic efficacy study..

[15] Iwelunmor J, Airhihenbuwa CO, King G, Adedokun A: Contextualizing Child Malaria Diagnosis and Treatment Practices at an Outpatient Clinic in Southwest Nigeria: A Qualitative Study.. ISRN Infectious Diseases,.

[16] Mohamed AH, Dalal W, Nyoka R, Burke H (2014.). Health care utilization for acute illnesses in an urban setting with a refugee population in Nairobi, Kenya: a cross-sectional survey.. BMC Health Serv. Res..

[17] Adeneye AK, Jegede AS, MafeM A, Nwokocha EE: (2013). Community perceptions and home management of malaria in selected rural communities of Ogun state, Nigeria.. International Journal of Malaria Research and Reviews:.

[18] Goodman C, Brieger W, Unwin A, Mills A (2007). Medicine Sellers and Malaria Treatment in Sub-Saharan Africa.. Am. J. Trop. Med. Hyg..

[19] Littrell M, Gatakaa H, Evance I, Poyer S (2011). Monitoring fever treatment behaviour and equitable access to effective medicines in the context of initiatives to improve ACT access: baseline results and implications for programming in six African countries.. Malar. J..

[20] Chuma J, Okungu V, Molyneux C: (2010). Barriers to prompt and effective malaria treatment among the poorest population in Kenya.. Malar. J..

[21] World Health Organization. Handbook: (2005). IMCI integrated management of childhood illness..

[22] NPC, Nigeria demographic and health survey. (2008). National Population Commission, Federal Republic of Nigeria,.

[23] National Population Commission (NPC) [Nigeria], National Malaria Control Programme (NMCP) [Nigeria], and ICF International. ((2012).). Nigeria Malaria Indicator Survey 2010..

[24] Zegers de Beyl C, Opawale A, Adegbe E, Baba E ((2011). Evaluation of the distribution campaign of Long‐Lasting Insecticidal Nets in December 2009, Niger State, Nigeria,.

[25] Gibbs, GR: (2002). Qualitative Data Analysis: Explorations with NVivo. Buckingham: Open University Press..

[26] Richards, L: (2010:). Handling Qualitative Data: A Practical Guide..

[27] Galman, SC: (2007). Shane, The Lone Ethnographer: A Beginner's Guide to Ethnography..

[28] Federal Ministry of Health. (2011)).

[29] Udoh E, Oyo-ita A, Odey F, Effa E (2013). Management of Uncomplicated Malaria in Under fives in Private and Public Health Facilities in South-Eastern Nigeria: A Clinical Audit of Current Practices.. Malaria Res. Treat..

[30] Uneke CJ: (2008). Concurrent malaria and typhoid fever in the tropics: the diagnostic challenges and public health implications.. J. Vector Borne Dis..

[31] Reyburn H, Mbatia R, Drakeley C, Carneiro I (2004). Overdiagnosis of malaria in patients with severe febrile illness in Tanzania: a prospective study.. BMJ..

[32] Källander K, Hildenwall H, Waiswa P, Galiwango E (2008). Delayed care seeking for fatal pneumonia in children aged under five years in Uganda: a case-series study.. Bull. World Health Organ..

[33] Clare IR, Chandler CI, Jones C, Boniface G (2008). Guidelines and mindlines: why do clinical staff over-diagnose malaria in Tanzania? A qualitative study.. Malar. J..

[34] Chinkhumba J, Skarbinski J, Chilima B, Campbell C (2010). Comparative field performance and adherence to test results of four malaria rapid diagnostic tests among febrile patients more than five years of age in Blantyre, Malawi.. Malar. J..

